# Rumen Fermentation Characteristics Require More Time to Stabilize When Diet Shifts

**DOI:** 10.3390/ani11082192

**Published:** 2021-07-23

**Authors:** Qinghua Qiu, Chaoyu Gao, Huawei Su, Binghai Cao

**Affiliations:** 1 State Key Laboratory of Animal Nutrition, College of Animal Science and Technology, China Agricultural University, Beijing 100193, China; rcauqqh@cau.edu.cn (Q.Q.); gaochaoyu@cau.edu.cn (C.G.); 2 Animal Nutrition and Feed Safety Innovation Team, Jiangxi Province Key Laboratory of Animal Nutrition/Engineering Research Center of Feed Development, College of Animal Science and Technology, Jiangxi Agricultural University, Nanchang 330045, China

**Keywords:** digestibility, rumen fermentation characteristics, sampling frequency, serum metabolism, stabilization time

## Abstract

**Simple Summary:**

Previous study revealed that the rumen bacterial community was in temporal dynamics, even after an adaptation of three months when diet shifted, while the dynamic rumen bacterial community is not necessarily in accord with varied rumen fermentation characteristics. Thus, no proper time for practical sampling frequency is available for conducting basal nutritional research in the long-term fattening stage of steers. This study aimed to evaluate the proper time for nutrient apparent digestibility, serum metabolic parameters, and rumen fermentation characteristics to stabilize when diet shifts. Results showed that nutrient apparent digestibility and serum metabolic parameters were stable across each collection month, while most rumen fermentation characteristics were affected by the interaction effects between collection period and dietary density. These results indicate that rumen fermentation characteristics require more time to stabilize when diet shifts, and it is recommended to collect ruminal digesta monthly to evaluate rumen fermentation characteristics.

**Abstract:**

This study was conducted to explore the proper time required to achieve stabilization in digestibility, serum metabolism, and rumen fermentation characteristics when different diets shift, thus providing decision-making of practical sampling frequency for basal nutritional research. For these purposes, 12 Holstein steers (body weight 467 ± 34 kg, age 14 ± 0.5 months) were equally assigned to two dietary treatments: high-density (metabolizable energy (ME) = 2.53 Mcal/kg and crude protein (CP) = 119 g/kg; both ME and CP were expressed on a dry matter basis) or low-density (ME = 2.35 Mcal/kg and CP = 105 g/kg). The samples of feces, serum, and rumen contents were collected with a 30-day interval. All data involved in this study were analyzed using the repeated measures in mixed model of SPSS. Results showed that nutrient apparent digestibility and serum metabolic parameters were stable across each monthly collection, while most rumen fermentation characteristics, namely concentrations of acetate, propionate, isobutyrate, and valerate, were affected by the interaction effects between collection period and dietary density. These findings indicate that rumen fermentation characteristics require more time to stabilize when diet shifts. It is recommended to collect ruminal digesta monthly to evaluate rumen fermentation characteristics, while unnecessary to sample monthly for digestion trials and blood tests in the long-term fattening of Holstein steers. This study may provide insights into exploring the associations between detected parameters and stabilization time, and between diet type and stabilization time when diet shifts.

## 1. Introduction

Nutritional studies with cattle are time-consuming and costly [1]. Health and biological functioning of livestock are often prioritized, and adaptation is necessary for animals to maintain metabolic balance when they are in a transition period [2,3]. An adaptation period is also needed in order to obtain representative samples for determination of nutrient apparent digestibility, serum metabolic parameters, and rumen fermentation characteristics, and is widely used in practical research of ruminants [4,5]. In the conventional beef fattening strategy of China, the fattening process is long and is generally divided into two or more stages (e.g., early–middle–late stage of fattening, early–late stage of fattening), with each stage keeping the same diet for several months [6]. Evaluation of the acceptable sampling frequency in each fattening stage could save both time and labor without detriment to the representativeness of detected samples, and assessment of the minimum time to achieve stability is an intuitive approach to determine the adaptation period.

Basal nutritional research with finishing cattle generally involves digestion trials to evaluate digestibility, blood tests to assess nutrition and health status, and ruminal digesta determination to monitor fermentation. Nicholson et al. [7] found the optimum period for the preliminary feeding period of steers in digestibility was 16 to 30 days, and they also emphasized that longer preliminary feeding periods would be needed when dietary density fluctuated widely. However, White et al. [8] stated that there was no necessity to extend the adaptation time to over 4 days in steers, since coefficients of variation for nutrient digestibility did not decrease appreciably after 4 days. Machado et al. [1] reported that the required time for stabilization varied with detected parameters when diet shifted, such as 9–13 days for voluntary intake, 6–13 days for ruminal digesta composition, and 4–11 days for ruminal fermentation characteristics. Sun et al. [9] observed daily dynamics in ruminal pH value, concentrations of lactic acid, total volatile fatty acids (TVFA), acetate, and propionate until 14 days after switching to a higher proportion of concentrate diet. Weimer et al. [10,11] found that lactation cows re-established their bacterial communities by 10 days after an exchange of rumen contents, despite the fact that ruminal bacterial community showed host specificity when the dairy cow was challenged with ruminal contents exchange. Our previous study [12] in finishing steers found that the rumen bacterial community was in dynamics, even after an adaptation of three months, when diet switched. Besides, Palmonari et al. [13] reported that ruminal pH values varied even in the similar bacterial communities. Therefore, dynamic rumen bacterial community is not necessarily in line with different rumen fermentation characteristics. More studies are still needed to uncover the acceptable and practical sampling frequency of finishing cattle in basal nutritional trials.

Two widely used density diets (high energy and high protein vs. low energy and low protein) in China were selected in this study to investigate: (1) whether samples collected monthly differ in digestibility, serum metabolic parameters, and rumen fermentation characteristics; (2) whether this difference or similarity was influenced by dietary nutrient density, thus providing decision-making for practical sampling frequency of finishing cattle in the early fattening stage with various density diets.

## 2. Materials and Methods

The animals’ care and experimental procedures were reviewed and approved by the China Agricultural University Animal Care and Use Committee (Permit No. AW09059102-2).

### 2.1. Experimental Design and Sample Collection

A total of 12 Holstein steers (body weight 467 ± 34 kg, age 14 ± 0.5 months) were equally allocated to two groups based on similar body weight (466.3 vs. 466.9 kg). The power analysis was performed using Power Analysis & Sample Size (PASS, Version 15.0.5, Kaysville, UT, USA) to check whether the sample size was enough and the power of the current sample size (*n* = 12) was 0.9985 based on alpha of 0.05. These steers were checked for parasitic infections and the results were all negative. All steers were fed the same pre-trial diet for four months before the start of the trial, and then shifted to either a high-density (H) or low-density (L) diet referring to National Academies of Sciences, Engineering, and Medicine (NASEM) [14]. H and L diets were metabolizable energy (ME, 2.53 vs. 2.35 Mcal/kg), crude protein (CP, 119 vs. 105 g/kg), neutral detergent fiber (NDF, 371 vs. 464 g/kg), and acid detergent fiber (ADF, 202 vs. 258 g/kg) under the same feedstuffs. The dietary density of the pre-trial diet was in the middle of the H and L diets to keep the same shifted nutrition level. A detailed description of ingredients and nutrient composition is listed in Table 1, also seen in our previous study [12]. Steers were housed in individual stalls. The experimental period lasted 90 days, and steers were fed *ad libitum* twice a day at 7:30 and 16:30, and had free access to fresh water. Here, we designated a month as the sampling interval to reduce sampling stress on rumen microorganisms, because many studies [15,16,17,18,19,20] have revealed that stress has a strong impact on rumen bacteria and may further affect rumen fermentation. During the whole experimental period, the temperature fluctuated within a range of −2.8 to 3.0 °C, and the relative humidity fluctuated within a range of 28.8 to 37.1%. The average daily gain across the experiment was 1.17 kg/day and 0.95 kg/day in H and L groups, respectively. Further basic growth performance of steers over three collection periods in high- and low-density diets are shown in Table S1 of Supplementary Materials.

**Figure 1:**
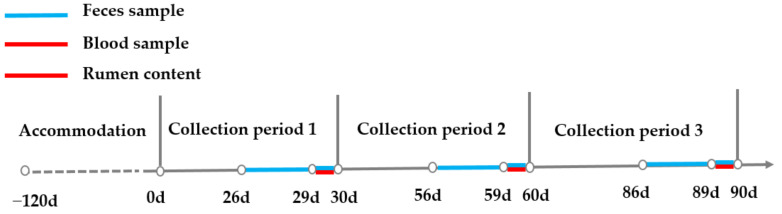
Illustration of sampling procedures. Notes: d indicates day and 0 d was the start of experiment.

Feed intake and orts were recorded daily at the same time point, and 110% of expected intake was given to assure the dry matter intake was not limited. Fecal samples of each steer were collected for five consecutive days at the end of each month, with the method of spot sample, and a sample interval of six hours for the first four days and interval of three hours on the last day from rectum. Time points on day 1 and 3 (00:00, 06:00, 12:00, 18:00), day 2 and 4 (03:00, 09:00, 15:00, 21:00), and day 5 (00:00, 03:00, 06:00, 09:00, 12:00, 15:00, 18:00, 21:00) were selected to guarantee a 3–day sample taken at 3–h intervals. Feces from 24 sampling time points of each steer were equally mixed into one sample for the monthly analysis. Blood was collected at the root of the tail one hour before morning feeding (07:30) on two consecutive days 29–30, 59–60, and 89–90 with a plain tube (Jiangsu Kangjian Medical Supplies Co., Ltd., Taizhou, China) without anticoagulation, and serum samples were obtained via centrifuging at 4000 rpm for 20 min. Rumen contents were collected three hours after morning feeding on two consecutive days 29–30, 59–60, and 89–90 using esophageal tubing, as described by Paz et al. [21], with both solid and liquid fractions collected by the same technician with the same procedure. Additionally, the first 200 mL was discarded to minimize saliva contamination [22]. A graphical description of sampling procedures is shown in Figure 1.

### 2.2. Chemical Analysis and Nutrient Apparent Digestibility Calculation

The analyzed nutrients in feeds, orts, and feces included dry matter (DM), organic matter (OM), CP, ether extract (EE), NDF, and ADF. DM content was obtained by oven drying at 105 °C for 8 h, OM was the nutrient combusted at 575 °C for 12 h, and CP and EE content were determined by Kjeldah method and Soxhlet extraction, respectively. The above nutrients were determined according to AOAC [23] using method 934.01, method 990.03, method 920.39, and method 942.05, respectively. NDF and ADF were analyzed by detergent of lauryl sodium sulfate and hexadecane trimethyl ammonium bromide, respectively, as Van Soest et al. [24] described, and the NDF was assayed with a heat-stable alpha-amylase (ANKOM FAA, ANKOM Technology Corp., Macedon, NY, USA) and expressed inclusive of residual ash.

The nutrient apparent digestibility was calculated according to Niu et al. [25], with acid insoluble ash (AIA) as the endogenous indicator to estimate the total fecal excretion. The calculation formula is shown as follows:

AD = [1 − (Ad × Nf)/(Af × Nd)] × 100%

where Ad (g/kg) and Af (g/kg) represent the AIA content of the diet and feces, respectively, and Nd (g/kg) and Nf (g/kg) represent the nutrient contents of the diet and feces, respectively. The evaluated nutrient apparent digestibility in this study included DM, OM, CP, EE, NDF, and ADF, and the values were expressed as g/kg.

### 2.3. Serum Metabolic and Ruminal Fermentation Parameters Determination

Serum biochemical parameters, including albumin, alkaline phosphatase, alanine aminotransferase, aspartate aminotransferase, cholesterol, creatinine, glucose, high-density lipoprotein cholesterol, low-density lipoprotein cholesterol, triglyceride, total protein, and urea N, were determined on the fifth day after each blood sampling using corresponding kits purchased from Beijing Jiuqiang Bio-Technique Co. via an automatic biochemical analyzer (Hitachi 7020, Hitachi Co., Tokyo, Japan). Ruminal fermentation parameters included ruminal pH value, volatile fatty acids (VFA), and ammonia nitrogen (NH_3_-N). The former was determined by a pH meter (Testo 205, Testo AG, Schwarzwald, Germany) immediately after collection. Ruminal contents were centrifuged at 20,000× *g* for 20 min at 4 °C, and then 200 µL metaphosphoric acid (250 g/L) and 800 µL supernatant were mixed evenly for determination of VFA, with 2-ethylbutyric acid (2.17 mL/L) as an internal standard. The VFA were analyzed by a gas chromatograph (GC-2014 Shimadzu Corporation, Kyoto, Japan) equipped with a 30 m polyethylene glycol-packed capillary column (Rtx-Wax, 0.25 mm ID × 0.25 µm film, Restek, Evry, France), using scheduled procedures in our previous studies [26,27]. The injection volume and injector temperature were controlled at 0.4 µL and 220 °C, respectively. The oven program was set as follows: initial temperature of 110 °C for 30 s, rise to 120 °C at a constant rate of 10 °C/min and then hold for 4 min, and gradually up to 150 °C in 3 min, during which the split ratio was controlled at 20:1 and the flow rate was kept at 2.5 mL/min. The individual component of VFA was identified according to the relative retention time of the standard curve, which was performed under the same condition and environment, with 2-ethylbutyric acid as the internal standard and respective VFA as the analytical standard, purchased from Sigma-Aldrich Limited Liability Company (Merck KGaA, Darmstadt, Germany). NH_3_-N concentration was determined according to Broderick and Kang [28] using a reader (Tecan, Mannedorf, Zurich, Switzerland), with the method of phenol-hypochlorite reaction.

### 2.4. Statistical Analysis

The Shapiro–Wilk test was conducted to confirm whether datasets of the current study conformed to normal distributions. All data were observed with normal distributions and did not require any transformation before analysis. All data in this study were analyzed using the repeated measures in mixed model of SPSS (version 20, IBM Corporation, Armonk, NY, USA). The statistical model is expressed as follows: y_ijt_ = μ + α_i_ + γ_t_ + (αγ)_it_ + c_j_ + e_ijt_, where y_ijt_ is the value of detected parameters, μ is the overall mean, α_i_ is the fixed effect of dietary nutrient density (i = 1, 2), γ_t_ is the fixed effect of collection period (t = 1, 2, 3), (αγ)_it_ is the interaction effect between dietary nutrient density and collection period, c_j_ is the random effect of cattle, and e_ijt_ is the residual effect. Comparison of means was conducted using Tukey tests with 95% confidence intervals (*p* < 0.05), and was marked with lower-case letters within the same row. A trend is defined when *p* values are between 0.05 and 0.10.

## 3. Results

### 3.1. Nutrient Apparent Digestibility

The nutrient apparent digestibility over three collection periods in H and L diets is shown in Table 2. No interactions between dietary nutrient density and collection period were observed in the apparent digestibility of DM, OM, CP, EE, NDF, and ADF (*p* > 0.10). The apparent digestibility of EE was higher in H diet than that in L diet (*p* < 0.05). The apparent digestibility of NDF (*p* = 0.059) and ADF (*p* = 0.077) tended to be higher in L diet than that in H diet. No differences were observed in nutrient apparent digestibility due to collection period, except for an increase in the apparent digestibility of OM (*p* = 0.003).

### 3.2. Serum Metabolic Parameters

Serum metabolic parameters over three collection periods in H and L diets are presented in Table 3. There were no interactions between dietary nutrient density and collection period regarding these detected metabolic parameters (*p* > 0.10). The concentrations of alanine aminotransferase, creatinine, and triglyceride were higher in L diet than that in H diet, while inverse in the concentration of urea N (*p* < 0.05). Increased trends were observed in H diet compared with L diet regarding aspartate aminotransferase to alanine aminotransferase ratio (*p* = 0.099), cholesterol (*p* = 0.056), high-density lipoprotein cholesterol (*p* = 0.065), and low-density lipoprotein cholesterol (*p* = 0.093). No differences were observed among collection periods, except for an increased trend in concentration of cholesterol (*p* = 0.069).

### 3.3. Rumen Fermentation Characteristics

Rumen fermentation characteristics over three collection periods in H and L diets are presented in Table 4. The concentration of acetate increased as collection period extended in H diet, whereas the opposite trend was observed in L diet (interaction, *p* < 0.001). Propionate, valerate, and TVFA concentrations increased as collection periods advanced in H diet, whereas no significant differences were observed in L diet regarding the above characteristics (interaction, *p* < 0.01). The concentration of total branched-chain volatile fatty acids (TBCVFA) decreased as collection periods extended in L diet; however, no significant differences were observed in H diet (interaction, *p* < 0.05). Isovalerate concentration tended to be affected by the interaction between collection period and dietary density (*p* = 0.068). Rumen pH and acetate to propionate ratio (A/P) were lower in H diet than that in L diet (*p* < 0.05). The A/P was gradually decreased as collection period advanced (*p* = 0.004).

## 4. Discussion

The steers used in this study were at their growth and fattening stage, during which feed intake (10.61, 10.81 and 11.27 kg/day in the first, second and third month, respectively) increased as months advanced to maintain increased body weight (502.4, 526.9 and 562.0 kg in the first, second and third month, respectively) [14], as shown in Table S1. Therefore, it is of no significance to take feed intake into consideration when exploring sampling frequency of finishing cattle. Total nutrient intake directly related to feed intake under the same dietary density. Thus, it is unnecessary to compare total nutrient intake across different months in finishing steers when evaluating sampling frequency. The apparent digestibility is a reflection of animals’ ability to digest nutrient, associated with both nutrient intake and fecal excretion [29]. The apparent digestibility of OM increased in the third collection period compared with that in the first collection period; this may be explained by the longer retention time of digesta in the third collection period. Because many studies [30,31,32,33] reported that digesta retention time increased as ambient temperature increased, average temperature was −1.2 °C and 3.0 °C in the first and third collection period, respectively. However, the absolute temperature and temperature differences of aforementioned reports were higher than the current study, suggesting that more studies were needed to uncover the definite temperature variation on rumen retention time and digestibility. In this study, no differences were observed in the apparent digestibility of DM, EE, CP, NDF, and ADF, indicating that an adaptation of one month is enough for preliminary feeding in digestion trials. These non-significances may be explained by the finding that the rumen bacterial community could return to pre-exchange communities by 10 days [10], and rumen microbes were associated with digestion [34]. These results were inconsistent with Lloyd et al. [35], who reported that digestibility values varied daily, even with an adaptation of 60 days. These inconsistencies may be due to the similarity between pretrial diet and H and L diets in the present study, as Nicholson et al. [7] showed that adaptation period extended as dietary hay to grain ratio varied. The close-spaced differences of dietary density between H and L diet may also account for the non-significance, which was designed to keep the consistency with practicability for early fattening stage of Holstein steers.

Blood biochemical parameters are effective indexes to monitor nutritional status and to diagnose disease [36], and many studies have revealed the age-dependent variations in metabolic parameters of cattle [36,37]. Blood biochemical values in this study were in the normal range of cattle with reference to Kaneko et al. [38], suggesting steers in the current condition were in good health and in line with animal welfare. The urea N is the main end-product of protein hydrolysis and amino acid metabolism [39], and the serum urea N is reported to be positively correlated with the dietary intake of crude protein [40]. These findings explain well the higher concentration of urea N in H diet than that in L diet, due to the higher daily protein intake in H diet (Table S1). The creatinine concentration reflects protein metabolism and a higher concentration of creatinine could be taken as an indicator of more muscular mass, leanness, and less feed efficiency during the finishing stage [40]. Our result showed higher creatinine in L diet than that of H diet, which suggests that steers of H diet gain a better growth rate, as revealed by higher average daily gain (Table S1). All of the detected biochemical parameters were not affected by collection period, nor by the interaction between dietary density and collection period; this could be explained partly by the experimental design in diets with small span in nutrient density. It is also possible that Holstein steers, with an age over 14 months old, possess stable serum metabolic characteristics in the same diet, because Mohri et al. [37] stated that many serum biochemistry parameters (albumin, alkaline phosphatase, aspartate aminotransferase, bilirubin, creatinine, globulin, and total protein) varied before puberty. The concentration of cholesterol tended to be higher in H diet than that in L diet. Possible explanations for this elevation may due to the higher apparent digestibility and higher content of ether extract in H diet (Table 2 and Table 1, respectively), and it is tempting to assume that serum cholesterol concentration is associated with the apparent digestibility of EE.

VFA are the main form of energy utilization in ruminants, and are known to play pivotal roles in production, growth performance, and product composition of ruminants [26,41]. Interactions between collection period and dietary density were observed in the concentrations of propionate, valerate, TVFA, and acetate. Results showed that the first three increased as collection periods advanced in single H diet, and the last one differed in inconsistent trends as collection period extended in H and L diets. These results indicated that the stabilization time for rumen fermentation characteristics varied with diets. Sutton et al. [42] found that propionate production, rather than acetate and butyrate production, had a more pronounced change when shifting to a low-roughage diet. Our study found that both propionate and acetate varied pronouncedly as collection period advanced in H diet. This result could be explained as fermentation of non-structural carbohydrates produced more propionate [41], and H diet was higher in starch content than the pre-trail diet (348 vs. 304 g/kg of DM). Additionally, most individual VFA, except for acetate and butyrate, altered as collection periods advanced in H diet, rather than in L diet. These results indicated that ruminal fermentation parameters adapted slower to a high-density diet. Comparing with Anderson et al. [43], who reported that the rumen bacterial community adapted faster to a high-density diet, it can be speculated that the rumen bacterial community and ruminal fermentation characteristic should be treated differently when evaluating adaptation period. Weimer et al. [10] reported that the bacterial communities of lactation cows could return to their previous profile by 10 days after rumen contents were exchanged, and the ruminal VFA composition and pH value recovered within 1 day. However, a report in growing steers showed that an adaptation period of two months was not enough to reach a stable bacterial community [44]. These reports indicated that age or physiological stage may also be a factor affecting adaptation period. Dijkstra et al. [41] reported that fermentation of protein produced more branched-chain VFA, such as isobutyrate and isovalerate. In this study, the difference in protein between pre-trial diet and shifted diet was small (7 g/kg, Table 1), and the difference in protein between H and L diet was larger (14 g/kg, Table 1). Therefore, it is reasonable to expect the significant and trending interactions between collection period and dietary density in the concentration of isobutyrate and isovalerate, respectively. Generally, structural carbohydrates ferment mainly into acetate, and nonstructural carbohydrates produced more propionate [41]. The acetate to propionate ratio (A/P) reflects the rumen fermentation pattern and is one of the indicators of health status [34]. The A/P in the current study were greater than 2.2, a threshold value of negative effects on the health and production performance of cattle [45,46], suggesting that steers were not suffering from health threat. It is unexpected to see that A/P decreased as collection period advanced in both H and L diets, because L diet was shifted from a diet with higher starch content to a lower one (304 vs. 259 g/kg of DM). Golder et al. [47] reported that a high starch diet decreased the abundance of *Fibrobacteres* and had a lower A/P, and Liu et al. [48] have also revealed that higher abundance of *Prevotella* contributed to higher propionate production. Qiu et al. [12] found increased relative abundances of *Fibrobacteres* (from 1.27 to 3.57%) and *Prevotella* (from 23.45 to 30.80%) as collection period advanced in the L diet. Perhaps the changes in bacterial community abundances responsible for amylolysis (7.35%) varied pronouncedly compared to those for fiber degradation (2.30%). However, to what extent they affect the production of propionate and acetate, respectively, remains unknown. Further research is needed to uncover the relationship between fermentation pattern and certain bacterial composition.

It should be admitted that the current research designed a month as the collection interval, and results showed that no differences among sampling periods occurred in nutrient apparent digestibility and blood biochemical parameters, which made it hard to know the exact days within 30 days needed to achieve stabilization regarding nutrient apparent digestibility and biochemical parameters. It was a pity that no samples on day 0 were collected, for considering that all steers were managed in the same mode for four months, and a more persuasive argument would be obtained if basal parameters were provided.

## 5. Conclusions

In summary, nutrient apparent digestibility and serum metabolic parameters were stable monthly, while most rumen fermentation characteristics were in dynamics as the month extended when diet shifted. Additionally, most individual VFA were affected by the interaction between diet and collection period. These results suggest that rumen fermentation characteristics require more time to stabilize when diet shifts, and it is recommended to collect ruminal digesta monthly to evaluate rumen fermentation characteristics, while unnecessary to sample monthly for digestion trials and serum biochemical parameters in the long-term fattening of steers. This study may also provide insights into exploring the associations between detected parameters and stabilization time, and between diet type and stabilization time when diet shifts.

## Figures and Tables

**Table 1:** Ingredients and nutrient composition of the experimental and pre-experimental diets.

Item		Diets ^1^	
	H	L	P
Ingredients, g/kg of DM			
Corn grain	414.4	299.4	356.9
Wheat grain	57.31	41.41	49.36
Soybean meal	73.63	53.20	63.42
*Leymus chinensis* ^2^	437.8	593.8	515.8
Calcium carbonate	5.62	4.06	4.84
Sodium chloride	5.62	4.06	4.84
Vitamin–mineral premix ^3^	5.62	4.06	4.84
Nutrient composition, g/kg of DM			
Dry matter (DM), g/kg	91.07	91.93	91.50
Organic matter (OM), g/kg of DM	94.89	94.66	94.78
Metabolizable energy (ME) ^4^, Mcal/kg of DM	2.53	2.35	2.44
Crude protein (CP)	119	105	112
Neutral detergent fiber (NDF)	371	464	418
Acid detergent fiber (ADF)	202	258	230
Acid detergent lignin (ADL)	31.4	38.3	34.8
Ether extract (EE)	32.5	30.5	31.5
Starch	348	259	304
Calcium	4.86	5.01	4.94
Phosphorous	2.59	2.32	2.46

Notes: ^1^ H denotes high-density diet, L denotes low-density diet, and P denotes pre-experimental diet. ^2^ *Leymus chinensis* was given as hay. ^3^ Premix provided the following per kg of dry matter (DM): 1.5 mg of vitamin A, 75 µg of vitamin D3, 45 mg of vitamin E, 30 mg of monensin, 60 mg of Fe, 63 mg of Zn, 99 mg of Mn, 200 mg of Cu, 0.5 mg of Se, 1.1 mg of I, 0.45 mg of Co, 877.4 g of rice bran. ^4^ Estimated as ME = total digestible nutrients × 0.04409 × 0.82.

**Table 2:** Nutrient apparent digestibility over three collection periods in high- and low-density diets.

Digestibility, g/kg	H Diet ^1^			L Diet ^2^			SEM ^3^	*p*-Value ^4^		
	HP1	HP2	HP3	LP1	LP2	LP3		Diet (D)	Period (P)	D × P
Dry matter (DM)	639.3	678.9	698.1	623.0	676.1	695.7	32.7	0.793	0.124	0.968
Organic matter (OM)	615.4	675.7	689.4	597.7	666.4	684.5	21.0	0.545	0.003	0.951
Crude protein (CP)	673.6	654.3	668.5	642.9	681.3	670.9	29.5	0.986	0.917	0.621
Ether extract (EE)	625.4	706.0	723.3	598.1	645.8	624.1	34.5	0.035	0.125	0.587
Neutral detergent fiber (NDF)	625.5	673.0	704.5	705.7	749.2	733.5	38.6	0.059	0.342	0.764
Acid detergent fiber (ADF)	620.4	670.7	700.9	696.4	742.2	726.9	38.7	0.077	0.312	0.776

Notes: ^1^ H = high-density diet, HP1 = samples of the first month of H diet, HP2 = samples of the second month of H diet, HP3 = samples of the third month of H diet. ^2^ L = low-density diet, LP1 = samples of the first month of L diet, LP2 = samples of the second month of L diet, LP3 = samples of the third month of L diet. ^3^ SEM = standard error of means. ^4^ D × P indicates interaction effects between dietary density and collection period.

**Table 3:** Serum metabolic parameters over three collection periods in high- and low-density diets.

Item ^1^	H Diet ^2^			L Diet ^3^			SEM ^4^	*p*-Value ^5^		
	HP1	HP2	HP3	LP1	LP2	LP3		Diet (D)	Period (P)	D × P
Albumin, g/L	31.2	29.7	32.3	33.8	31.8	31.7	1.11	0.168	0.334	0.287
Alkaline phosphatase, U/L	116	96.6	103.2	94.8	81.3	89.6	16.1	0.249	0.684	0.979
ALT, U/L	18.4	20.5	19.1	23.3	23.6	22.9	1.24	0.001	0.590	0.785
AST, U/L	33.4	28.0	35.5	38.8	36.4	39.6	2.77	0.260	0.872	0.408
AST/ALT	1.82	1.93	2.07	1.68	1.59	1.76	0.19	0.099	0.604	0.840
Cholesterol, mmol/L	2.51	2.56	3.12	2.31	2.49	2.50	0.17	0.056	0.069	0.189
Creatinine, μmol/L	63.8	58.6	66.8	71.3	69.5	76.0	4.08	0.021	0.162	0.935
Glucose, mmol/L	3.72	3.92	3.90	3.89	3.94	3.91	0.12	0.507	0.569	0.782
HDLC, mmol/L	1.07	1.06	1.17	0.97	1.04	1.03	0.05	0.065	0.365	0.476
LDLC, mmol/L	0.65	0.68	0.83	0.65	0.64	0.65	0.05	0.093	0.114	0.160
Triglyceride, mmol/L	0.15	0.19	0.20	0.26	0.28	0.29	0.02	<0.001	0.204	0.907
Total protein, g/L	48.5	48.9	47.8	52.7	46.5	49.9	2.20	0.485	0.439	0.313
Urea N, mmol/L	3.05	3.77	3.55	1.99	2.33	2.46	0.27	<0.001	0.225	0.742

Notes: ^1^ ALT, alanine aminotransferase; AST, aspartate aminotransferase; HDLC, high-density lipoprotein cholesterol; LDLC, low-density lipoprotein cholesterol. ^2^ H = high-density diet, HP1 = samples of the first month of H diet, HP2 = samples of the second month of H diet, HP3 = samples of the third month of H diet. ^3^ L = low-density diet, LP1 = samples of the first month of L diet, LP2 = samples of the second month of L diet, LP3 = samples of the third month of L diet. ^4^ SEM = standard error of means. ^5^ D × P indicates interaction effects between dietary density and collection period.

**Table 4:** Rumen fermentation characteristics over three collection periods in high- and low-density diets.

Item ^1^	H Diet ^2^			L Diet ^3^			SEM ^4^	*p*-Value ^5^		
	HP1	HP2	HP3	LP1	LP2	LP3		Diet (D)	Period (P)	D × P
Rumen pH	6.65	6.65	6.63	6.70	6.82	6.80	0.07	0.039	0.628	0.535
NH_3_-N, mg/dL	3.29	3.93	3.75	2.56	2.61	2.51	0.49	0.098	0.618	0.675
Acetate, mmol/L	42.4 ^c^	45.4 ^ab^	51.7 ^a^	52.0 ^a^	50.0 ^ab^	43.0 ^bc^	2.29	0.478	0.964	<0.001
Propionate, mmol/L	10.1 ^b^	11.8 ^ab^	14.0 ^a^	9.92 ^b^	10.8 ^b^	10.3 ^b^	0.58	0.041	<0.001	0.001
Isobutyrate, mmol/L	0.64 ^ab^	0.69 ^a^	0.63 ^ab^	0.56 ^bc^	0.52 ^c^	0.48 ^c^	0.02	0.001	0.006	0.010
Butyrate, mmol/L	6.01	6.67	7.16	5.48	7.01	6.40	0.45	0.469	0.059	0.407
Isovalerate, mmol/L	0.87	0.91	0.86	0.68	0.64	0.48	0.04	<0.001	0.014	0.068
Valerate, mmol/L	0.58 ^c^	0.71 ^ab^	0.76 ^a^	0.57 ^c^	0.62 ^bc^	0.56 ^c^	0.03	0.009	<0.001	0.001
TVFA, mmol/L	60.7 ^b^	66.2 ^ab^	75.1 ^a^	69.2 ^ab^	69.5 ^ab^	61.2 ^b^	3.05	0.849	0.296	<0.001
Acetate to propionate ratio	4.21	3.85	3.70	5.26	4.70	4.16	0.19	<0.001	0.004	0.338
TBCVFA, mmol/L	1.51 ^a^	1.60 ^a^	1.49 ^a^	1.24 ^b^	1.16 ^bc^	0.96 ^c^	0.06	<0.001	0.002	0.016

Notes: ^1^ NH_3_-N, ammonia nitrogen; TVFA, total volatile fatty acids, equal to the sum of acetate, propionate, isobutyrate, butyrate, isovalerate, and valerate; TBCVFA, total branched-chain volatile fatty acids, equal to the sum of isobutyrate and isovalerate. ^2^ H = high-density diet, HP1 = samples of the first month of H diet, HP2 = samples of the second month of H diet, HP3 = samples of the third month of H diet. ^3^ L = low-density diet, LP1 = samples of the first month of L diet, LP2 = samples of the second month of L diet, LP3 = samples of the third month of L diet. ^4^ SEM = standard error of means. ^5^ D × P indicates interaction effects between dietary density and collection period. ^a–c^: different superscripts indicate significant differences within a row (*p* < 0.05).

## Data Availability

Not applicable.

## Supplementary Materials

The following are available online at https://www.mdpi.com/article/10.3390/ani11082192/s1, Table S1: Growth performance of steers over three collection periods in high- and low-density diets.
