# Supplementary material for: Rumen Fermentation Characteristics Require More Time to Stabilize When Diet Shifts
Source: Animals (Basel). 2021 Jul 23;11(8):2192. doi: 10.3390/ani11082192 (PMC8388484; doi:10.3390/ani11082192)
Supplement: Supplementary file 1 [file animals-11-02192-s001.zip › animals-1255477-supplementary.pdf]

Table S1. Growth performance of steers over three collection periods in high- and low-density diets.

Item	H diet [1]			L diet [2]			SEM [3]	p – value [4]		
	HP1	HP2	HP3	LP1	LP2	LP3		Diet (D)	Period (P)	D × P
Initial body weight, kg		466.3			466.9		14.37		0.978	
Final body weight, kg	508.6	534.6	571.4	496.2	519.1	552.5	14.14	0.450	<0.001	0.305
Feed intake, kg/d	11.11ab	11.22ab	11.48a	10.11b	10.40ab	11.05ab	0.29	0.098	<0.001	0.001
Energy intake, Mcal/d	28.11a	28.39a	29.04a	23.77b	24.44b	25.96ab	0.72	0.004	<0.001	0.003
Protein intake, kg/d	1.32a	1.34a	1.37a	1.06b	1.09b	1.16b	0.03	<0.001	<0.001	0.004
Average daily gain, g/d	1409	865	1228	976	763	1114	87.85	0.049	0.002	0.101
Feed conversion ratio [5]	8.30	13.42	9.50	10.89	14.26	10.14	0.96	0.237	0.001	0.378

Notes:[1] H = high-density diet, HP1 = samples of the first month of H diet, HP2 = samples of the second month of H diet, HP3 = samples of the third month of H diet.[2] L = low-density diet, LP1 = samples of the first month of L diet, LP2 = samples of the second month of L diet, LP3 = samples of the third month of L diet. [3] SEM = standard error of means. [4] D × P indicates interaction effects between dietary density and collection period. [5] Feed conversion ratio was calculated as: feed intake (kg/d)/average daily gain (kg/d). a–b: different superscripts indicate significant differences within a row ($p < 0.05$).
